# Mr. Wood, on the Treatment of Burns and Scalds

**Published:** 1808-03

**Authors:** J. Wood

**Affiliations:** Newcastle


### *To the Editors of the Medical and Physical Journal.*

GENTLEMEN,

IN your Journal for July, 1806, you will find my intention expressed of soon making another communication to you, containing a Prospectus of an object I have long had in contemplation, together with some general principles, that would include the theory of the treatment of Burns and Scalds. That year, and another year is con-

 cluded, cluded, and a third has commenced, without my fulfilling my intention. Soon after I last addressed you, my mind, as far as it could be taken from professional duties, became wholly occupied by domestic sickness,* that terminated in a severe affliction in the beginning of last year, by which most of my plans have been superseded. I have thought this explanation necessary, as an apology due to you, and as a view of my silence due to my subject and myself.

The object to which I have alluded, has been, to ascertain what diseases, out of the number of those commonly treated by stimulants, &c. as the means of cure, were capable of being cured by opposite means. I began in 1791 with typhus fever, and soon fully satisfied myself that the refrigerating plan, or that which abstracted stimuli, instead of applying such, was the most successful and rational mode of cure. In 1793, I collected a few ideas on the subject, which, together with some general principles, I published in that year. To exemplify one of said general principles, I used the following words. "We can apply, not only with safety but with the best effect, ardent spirits to a part burned or scalded." Were it necessary, I could produce such circumstantial evidence as would be admitted even in a court of justice, that the above words were the origin of the stimulating plan of cure by Dr. Kentish in this place, and of his consequent publication on the subject. When that publication appeared, I knew that some of the first surgeons here pointed out *my hint* as the first they had received on the subject. It was never even alluded to by Dr. Kentish, though I then, and ever since, have openly maintained its priority. At the time I published those general principles, the emollient plan of cure was, I have always understood, universally used; cold applications were not, I believe, ever thought of.

It is said, that an irregular practitioner in this neighbourhood has long used spirituous applications in cases of burns; but at the time I exemplified my general principle, by the above instance of the efficacy of ardent spirits to parts burned or scalded, I had resided a very short time in this place, and was equally ignorant of the practice of the regular and irregular practitioner. A common desire for

* Any detail of this, though I trust it might be useful, would, if even my feelings allowed me to give it, be out of its place here. The result, shewing how hectic fever, with its usual causes present, may, in many instances, be almost wholly prevented, and life consequently prolonged, may probably be, at a future period, offered to the public.

for justice alone has induced me to make this public statement, which I doubt not I can maintain yet I by no means consider the value of the discovery (if it may be so called) of such importance as to be worth a competition. At the time that I gave the example, I was not satisfied that it was always the best practice; and it will now appear, from a farther explanation of those general principles which guide my practice, that in a certain state of inflammation I would never use any stimulating application, and that it is only when another state has commenced that I would think it necessary to have recourse to it.

In the publication I have mentioned, I observed, that, evidently, two kinds of inflammation took place in ophthalmia, requiring a very opposite treatment; but I was not *then* aware, that *one* kind of inflammation was *capable* of being cured by *two different* means of cure; or, to speak more plainly, I soon observed, that inflammation from debility could only admit of one method of cure, but that inflammation from too high action might be cured by two, and opposite methods of treatment. *This observation removed my first and greatest difficulty in my investigation, it solved easily questions before inexplicable, reconciled apparent contradiction, and, last of all, gave the best explanation of the origin of the adage, that, "Doctors differ."*

It explained how recovery in typhus took place, both under the refrigerating and the stimulating plan of treatment; how both plans were successful in burns and scalds, and, carried to a similar diseased action of the sensorium, how *fever of the brain* was cured by opposite means. In all such states I have been invariably successful by the application of *cold*, and the aid of purgatives; and yet I cannot but doubt such states have been cured by the use of stimulants. Although, then, common inflammation, typhus fever, brain fever, gout, and other similar states of diseased action, are capable of being cured by different, and nearly opposite means; yet it does not follow, that *both* are *equally* good, and that one has not the preference to the other. The refrigerating plan of treatment, or that of directly lessening diseased action, must always have the preference by every mind that is open to true philosophy, in every instance in which it can with safety be applied; it is not only the most simple plan, but the cure, when it can be performed by the cooling plan, is always obtained by less expence to the constitution, than when it is performed by the opposite means. From what I have now

 comcommunicated, you will be sensible, that I had entered into a wide field of investigation. To what advantage I have done so, the public, I hope, will have the means of judging more fully in the course of this year; I have too carried my inquiries into the different diseased states of the nervous system; and I trust I can explain, why chorea has been cured by Dr. Hamilton and others by purgatives, and why the *tonic* plan of treatment has in my practice been *invariably* successful; my general theory on this subject I shall have an opportunity of illustrating in another part of this communication.

These sentiments may appear to be too confidently expressed, but they arise not from the warm hopes of the theory of a day, but from the cool, well tried, and, I trust, mature experience of *sixteen years.*

When I hear of disputes on the best method of treating gout, burns and scalds, ophthalmia, rheumatism, &c. I desire a *given state* to be mentioned, and this saves the half at least, if not the whole of the dispute; in actual practice this will always require much penetration, and a nice judgment. It is, indeed, a great difficulty immediately to ascertain the *real state,* for when that is *given* with *certainty,* the means of cure easily follow; but if the wrong state is given, the means of cure may not only in one state be *opposite* to the *best,* but also, in another state, be *totally opposite* to what they should be, and positively hurtful. I have not at present had recourse much to the ideas and language of the late Dr. Brown. Although both my theory and practice is very different from his, yet my general principles are founded on his doctrine. The errors of his practice have been frequently exposed; and *in the very place* where both have been most condemned, *it is now admitted,* that part of his doctrine has simplified and improved our language in medicine.

After having expressed my intention of offering some general principles, that would include the theory of the treatment of burns and scalds, the discussion of the same subject by Dr. Kinglake and Mr. Harrold, could not fail to be interesting to me, especially as they both took for their foundation *nature,* or Dr. *Brown. From nature* and Dr. *Brown,* I have been rearing the fabric, which I have sometimes indulged myself with the hope, may be as perfect as the present state of our ideas will admit.

By mentioning in what point I differ from Dr. Kinglake, and in what from Mr. Harrold, it will not only be shewn how far *my* theory and practice are Brunonian, but also the principles that I have adopted will more plainly appear.

From

From Mr. Harrold I *differ* as to the mode of lessening increased action, or too much excitement; with him, and the Brunonian theory I *agree* as to the means of restoring the *state of direct debility* to that of health; but I am surprised, that because by both, it is considered necessary to apply stimulants, *in that state, mild* and *gradual* in *proportion* to the *degree* of *debility*, they should therefore think, that the *opposite* state of high excitement should be *also gradually* reduced. The *very reverse* should take place in the *mode* and the *means*, as has taken place in the *state*. I agree with Mr. Harrold therefore in differing with Dr. Kinglake, when he says, that whether the departure from the standard of health consists "in an excessive or deficient action of vital power, a prompt restoration of that standard is the eligible mode of cure." I also differ from Dr. Kinglake, when he says, that, "a *negative* condition of vital power cannot be *positively* active," because, I think I can make it appear, that inflammation from a *negative condition* of vital power is a *positive action*. Except in these two instances, I think that Dr. Kinglake's observations, respecting the actions of organic life, strongly coincide with those I have made, and his arguments, in every other, very forcible and conclusive; I mean, on the general subject. For what I have already stated of my particular ideas as to the propriety of cold applications in gout, or any inflammation, will sufficiently explain my sentiments. That there must be a *given* state, and a *given degree* of the *power* applied, before it can be said, whether the power is adapted to the state; for instance, the long continued application of cold may, by *directly* lessening action, be very effectual in removing ophthalmia from too great excitement, that is, a high action from strongly exciting causes in the full tone of health. We know that cold alone will *produce* ophthalmia, chilblains, &c. (commonly observed in the fibre of weak tone, or the scrofulous pre-disposition;) and, yet, cold again may in this ophthalmia be applied in *that degree*, which will make it act as a *tonic*, and cure the ophthalmia, by *indirectly* lessening action; that is, it *lessens action* in such a state, by *increasing tone*; or, perhaps, it increases tone at the same time that it lessens action. The different effects of the same degree of cold on different states, and the different effects of the same degree of cold on the same state, in illustration of this theory, are very well known. On the muscular fibre, not in a state of inflammation, but of debility, a certain degree of cold will excite inflammation by by its debilitating effects; the same degree of cold acts only as a tonic to a person in a state of health, while it proves the means, in some small degree, of lessening action in a state of great excitement; but that degree of cold, which acts only as a tonic in a state of debility, will be little felt by the body in a state of health, and have little or no effect in lessening high excitement.

As the remaining intended part of this communication has not any connection with the above subject, I think it will be better to send it to you for another Number of your Journal, especially as my leisure, at this time, will not conveniently admit of my making it a part of this Paper, as I proposed.

I am, &c.

J. WOOD.

*Newcastle, January* 20, 1808.

